# Monitoring Dynamics, Structure, and Magnetism of Switchable Metal–Organic Frameworks via ^1^H‐Detected MAS NMR

**DOI:** 10.1002/anie.202107032

**Published:** 2021-09-02

**Authors:** Jan Blahut, Arthur L. Lejeune, Sebastian Ehrling, Irena Senkovska, Stefan Kaskel, Florian M. Wisser, Guido Pintacuda

**Affiliations:** ^1^ Centre de Résonance Magnétique Nucléaire à Très Hauts Champs UMR 5082 CNRS ENS Lyon UCBL) Université de Lyon 69100 Villeurbanne France; ^2^ NMR Laboratory Faculty of Science Charles University Hlavova 8 12842 Prague Czech Republic; ^3^ IFP Energies Nouvelles 69360 Solaize France; ^4^ Chair of Inorganic Chemistry I Technische Universität Dresden 01069 Dresden Germany; ^5^ IRCELYON (UMR 5256 CNRS, UCBL) Université de Lyon 69100 Villeurbanne France; ^6^ Institute of Inorganic Chemistry University of Regensburg 93040 Regensburg Germany; ^7^ Present address: 3P Instruments GmbH & Co. KG Rudolf-Diesel-Strasse 12 85235 Odelzhausen Germany

**Keywords:** DUT-8(Ni), fast magic-angle spinning, metal–organic framework, paramagnetic NMR, proton detection

## Abstract

We present a toolbox for the rapid characterisation of powdered samples of paramagnetic metal–organic frameworks at natural abundance by ^1^H‐detected solid‐state NMR. Very fast MAS rates at room and cryogenic temperatures and a set of tailored radiofrequency irradiation schemes help overcome the sensitivity and resolution limits often associated with the characterisation of MOF materials. We demonstrate the approach on DUT‐8(Ni), a framework containing Ni^2+^ paddle‐wheel units which can exist in two markedly different architectures. Resolved ^1^H and ^13^C resonances of organic linkers are detected and assigned in few hours with only 1–2 mg of sample at natural isotopic abundance, and used to rapidly extract information on structure and local internal dynamics of the assemblies, as well as to elucidate the metal electronic properties over an extended temperature range. The experiments disclose new possibilities for describing local and global structural changes and correlating them to electronic and magnetic properties of the assemblies.

## Graphical abstract

Paramagnetic DUT‐8(Ni) metal–organic frameworks were characterised over an extended temperature range with ^1^H‐detected NMR techniques under fast magic‐angle spinning. The sensitivity of the approach allows rapid access to structure and dynamics of the organic linkers as well as the electronic properties of the metal centres in these materials.

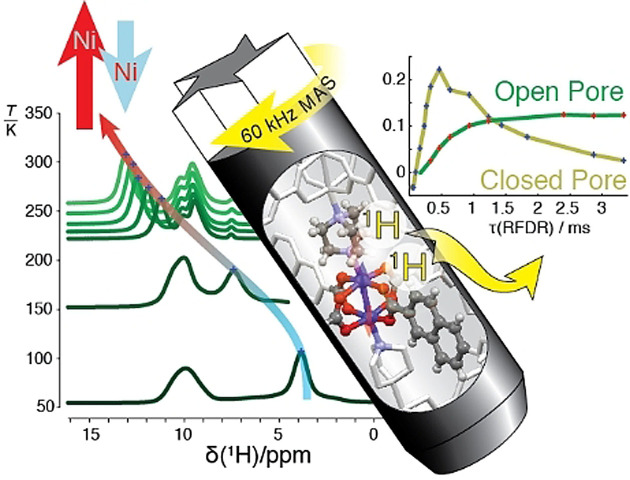

Crystalline porous coordination polymers or metal–organic frameworks (MOFs) represent a versatile family of materials with efficiently tunable properties by a combination of various metal‐based nodes with an inexhaustible plethora of organic ligands.[^1^, ^2^, ^3^, ^4^] The possibility to control the pore size and shape by design, as well as facile material functionalisation make MOFs attractive for adsorption‐based applications. Following the seminal report by Kitagawa,^[5]^ a large class of MOFs features highly flexible architectures, capable of undergoing structural changes dependent on the presence of guest molecules.^[6]^ These guest‐dependent transitions affect, in turn, the optical and magnetic properties of the materials, opening new perspectives for the use of MOFs in catalysis,^[7]^ gas separation,[^8^, ^9^, ^10^] or sensing.[^11^, ^12^, ^13^]

Atomic‐level characterisation of MOFs represents the essential link capable of explaining and controlling the macroscopic properties of these efficient materials in terms of their microscopic structures and dynamics.[^14^, ^15^, ^16^, ^17^] Given the difficulties associated with the growth of single crystals and their manipulation during guest adsorption, diffraction methods are significantly limited in this area.^[18]^ Solid‐state nuclear magnetic resonance (NMR) provides a powerful alternative, capable of interrogating the local structures around metals, the configurations of organic ligands, their dynamics and the environments experienced by guest molecules.[^19^, ^20^, ^21^, ^22^, ^23^, ^24^, ^25^, ^26^, ^27^, ^28^] Despite the tremendous recent technological and methodological advances, however, this technique still falls short in many aspects, as it requires large sample quantities, long experimental measurements and data analyses, and often complex isotopic labelling schemes.

The direct detection of ^1^H signals is the most obvious way to circumvent these difficulties by enhancing the sensitivity of the NMR experiments, due to the high gyromagnetic ratio *γ* of ^1^H spins, their natural isotopic abundance, and their ubiquitous presence in MOF organic constituents. In solids, however, the very same properties of ^1^H spins form a dense network of strong dipolar couplings, which broadens ^1^H resonances and hampers their constructive use.^[29]^ The direct detection of ^1^H resonances in MOFs has been the object of notable proof‐of‐concept reports,[^30^, ^31^, ^32^, ^33^, ^34^] but spectra often lacked the resolution required for the complete identification of individual ^1^H sites. The problem is alleviated by fast magic‐angle spinning (MAS) at high magnetic fields, which weakens the ^1^H dipolar coupling networks and sharpens their NMR lines.[^35^, ^36^] However, MAS rates achievable with commercially available NMR probes today are not sufficient to yield fully resolved ^1^H spectra of MOFs, and the only site‐resolved ^1^H‐detected NMR structural studies relied on ^1^H spin dilution involving (complex and costly) partial replacement of ^1^Hs with ^2^Hs, which significantly limits the advantages of the approach.^[37]^

MAS NMR of MOFs containing open‐shell metal ions carries an additional layer of experimental difficulties.[^38^, ^39^, ^40^, ^41^, ^42^] In these samples, the hyperfine interactions between NMR‐active nuclei and the unpaired electrons of the paramagnetic metals introduce important problems in the acquisition and interpretation of the spectra.^[43]^ At the same time, however, these interactions encode important information of the geometry and the electronic structure of the metal environments. Also in this case, fast MAS is a crucial factor for the detection of nuclei in close proximity to a metal centre.[^44^, ^45^] However, as some paramagnetic effects are larger on high‐*γ* ^1^H nuclei, MAS NMR of paramagnetic MOFs has up to now focused preferentially on low‐*γ* nuclei (^13^C, ^15^N, ^17^O, ^2^H, etc.) which suffer from low natural abundance with only limited applicability of isotope labelling.

Here, we show that with the help of very fast (60 kHz) MAS rates and a set of tailored radiofrequency (RF) irradiation schemes,[^46^, ^47^, ^48^, ^49^] paramagnetic effects can become an asset for the rapid characterisation of powdered samples of open‐shell MOFs at natural abundance by ^1^H‐detected solid‐state NMR. This includes sensitive detection and assignment of resolved ^1^H and ^13^C resonances of organic linkers via 2D ^1^H–^1^H and ^1^H–^13^C correlations, analysis of structure and local internal dynamics, as well as elucidation of the metal electronic properties over an extended temperature range.

We showcase this methodology on a prototypical switchable pillared layer MOF offering a variable metal substitution chemistry, namely DUT‐8(M) (DUT=Dresden University of Technology),^[50]^ composed of (M^2+^)_2_ paddle‐wheel (PW) units linked through 2,6‐naphthalenedicarboxylate (NDC) into 2D layers, which are in turn interconnected by 1,4‐diazabicyclo[2.2.2]octane (DABCO) pillars (Scheme 1). We notably focus on DUT‐8(Ni) containing (Ni^2+^)_2_ PWs, which exhibits strong hysteresis during guest adsorption/desorption between an open‐pore (**op**) including a guest molecule (DMF in this study) and a closed‐pore (**cp**) structure. This reversible, guest‐dependent structural change is associated with a significant reorientation of the DABCO pillars with respect to the PW plane (the Ni⋅⋅⋅Ni‐N angle changes from approx. 178° to 154°), which maintains the Ni⋅⋅⋅Ni distance almost unaltered but strongly deforms the square‐pyramidal coordination of the nickel and produces an unpreceded decrease (by 60 %) of the unit‐cell volume.[^50^, ^51^]

**Scheme 1:**
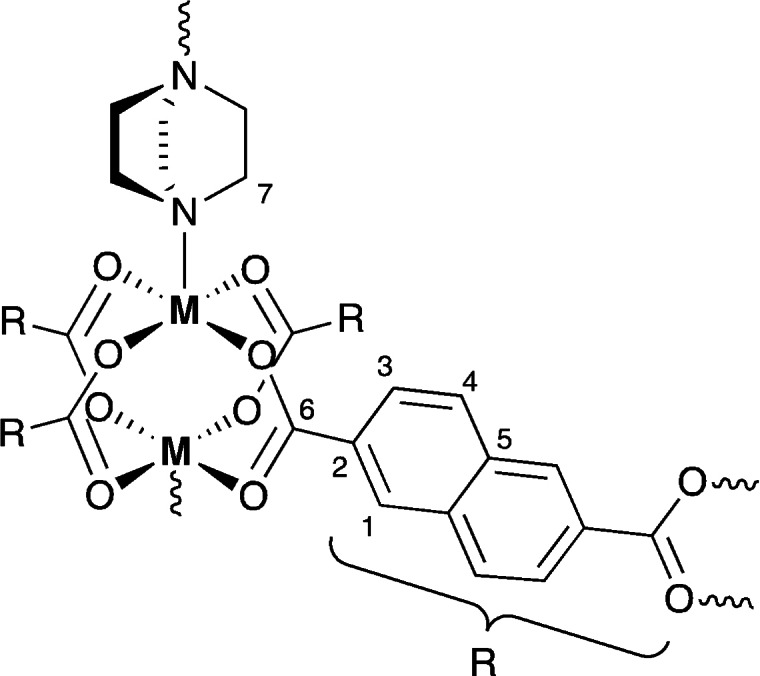
DUT‐8(M) with labelling used in the text.

Figure 1 A,B shows 1D ^1^H spin‐echo spectra of **cp** and **op** DUT‐8(Ni) powders, respectively, acquired on a 700 MHz spectrometer at 60 kHz MAS. These spectra illustrate the challenge associated with ^1^H detection and paramagnetic NMR in these paddle‐wheel‐based frameworks. Due to the combined effect of ^1^H–^1^H homonuclear interactions and hyperfine coupling with the unpaired electrons of the paramagnetic metal ions, the spectra contain highly overlapped centre‐band lines (inset for Figure 1 B) and a pronounced pattern of rotational sidebands. The richness of paramagnetic NMR effects nonetheless provides a way to monitor the dynamic structural transformation between the two states associated with distinct spectral responses. Both spectra are characterised by an unresolved or partially resolved set of lines (approx. 9.5 ppm) from the NDC ligands (H1/H3/H4) and shifted downfield a baseline‐resolved signal from the ^1^Hs in the DABCO pillars (H7). The latter, however, resonates at 13.5 ppm in the **op** form and at 48.8 ppm in the **cp** structure, which immediately reveals significantly different paramagnetic contributions, and thus different electronic structures and spin‐density distributions, in the two samples. Moreover, ^1^H longitudinal and transverse relaxation rates are similarly enhanced in the two spectra, but linewidths feature a ten‐fold difference in the two samples (e.g. 440 and 5600 Hz for the DABCO ^1^Hs (H7) in the **op** and **cp** sample, respectively, see Table S1). This effect is a clear signature of the presence of a large inhomogeneous broadening in the latter spectrum, compatible with a more diverse structure with frequent irregularities for the **cp** sample indicated by the powder X‐ray diffraction analysis.^[51]^ Finally, the very different chemical shift anisotropies (about twice as high for the **cp** structure) indicate largely different magnetic susceptibilities in the two samples, in line with an antiferromagnetic coupling between neighboring Ni^2+^ ions in the **op** structure (see below).

**Figure 1:**
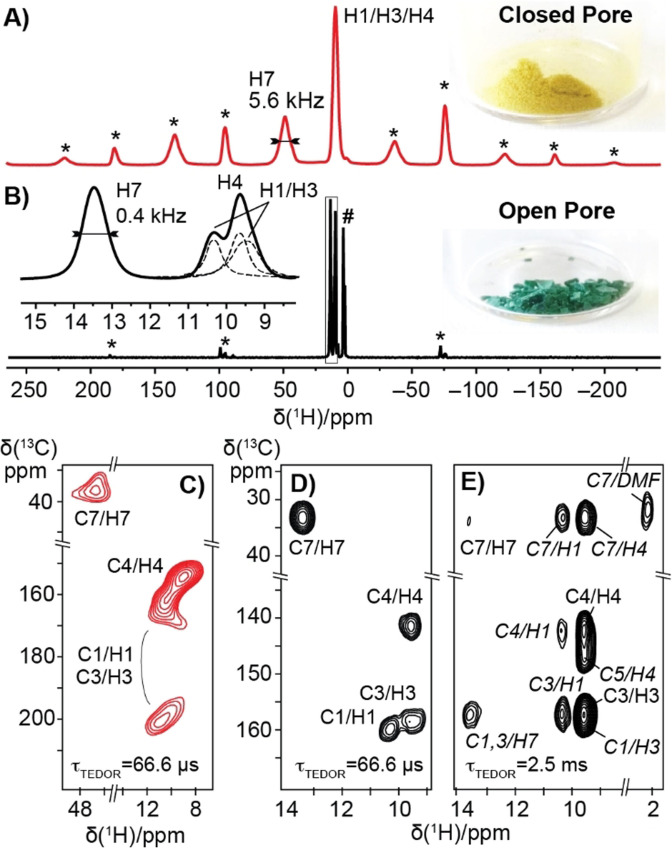
Solid‐state MAS ^1^H NMR spectra of A) **cp** and B) **op** DUT‐8(Ni) with expansion including deconvolution of the NDC spectral region (dashed line). Spectra acquired with rotor‐synchronised spin‐echo detection at 60 kHz MAS (700 MHz, 325 K of sample temperature). Rotational sidebands and residual solvent signals are indicated by an asterisk (*) and a hash mark (#), respectively. The displayed line‐width of the H7 resonance corresponds to the full‐width at half maximum. For the labelling of resonances, see Scheme 1. C–E) ^1^H–^13^C HSQC‐TEDOR spectra of **cp** (C, red contours) and **op** DUT‐8(Ni) (D,E, black contours), acquired with short (66.6 μs, C and D) and long (2.5 ms, E) *τ*_TEDOR_ recoupling. In (E) long‐range correlations are labelled in italics.

An unambiguous identification of all individual isotropic ^1^H resonances can be achieved by extending the ^1^H spin‐echo spectra into 2D ^1^H–^13^C correlations. While in diamagnetic samples the transfer of polarisation from protons to nearby hetero‐nuclei is performed routinely by cross‐polarisation, pulsed techniques such as the Transferred Echo DOuble Resonance recoupling (TEDOR)^[52]^ allow a more efficient magnetisation transfer for nuclei exposed to strong paramagnetic shift and fast paramagnetic‐induced relaxation.^[45]^ TEDOR uses rotor‐synchronised π pulses to reintroduce, during an interval *τ*_TEDOR_, the ^1^H–^13^C dipolar couplings otherwise averaged out by MAS, and is usually acquired as a ^13^C‐detected experiment at intermediate MAS rates in the 30 kHz range. Thanks to the improved resolution and the shorter recoupling periods possible at 60 kHz MAS, we adopted here a ^1^H‐detected variant (Heteronuclear Single‐Quantum Correlation through TEDOR or HSQC‐TEDOR, Figure S1). This scheme was recently proposed for the characterisation of the coordination sphere of a paramagnetic metalloenzyme,^[49]^ and provides comparable sensitivity to the ^13^C‐detected spectrum acquired at slower MAS, with a five‐fold reduction in the sample volume. The uniformly short *T*_1_ relaxation times, together with the absence of the requirement for high‐power RF irradiations during the experiment,^[44]^ allow here the use of very short recycle delays between acquisitions (5 ms). Combined with the high sensitivity of ^1^H detection, this produces a sensitivity boost, and a 2D correlation can be acquired within 30 minutes on 1–2 mg of sample at natural ^13^C abundance. Depending on the *τ*_TEDOR_ recoupling period employed, ^1^H–^13^C correlations can be observed either over short distances corresponding to a single bond (Figure 1 C,D), or over longer distances, including correlations between ^1^Hs and quaternary ^13^Cs (Figure 1 E). These TEDOR spectra resolve and assign the individual frequencies of all the ^1^H and ^13^C nuclei in the sample.

As illustrated in Figure 2, the sensitive detection of resolved ^1^H resonances represents a powerful handle for the characterisation of the framework architecture and its local dynamics at atomic resolution. Figure 2 A,B shows an example of 2D ^1^H–^1^H correlations obtained with Radio‐Frequency‐Driven Recoupling (RFDR),^[54]^ as well as the build‐up curve of the cross‐signals between NDC and DABCO ligands. This experiment plays a similar role as the ^1^H–^1^H NOESY in solution, where the dependence of the volume of ^1^H–^1^H cross‐signals with the mixing time as a function of ^1^H–^1^H distances forms the basis of NMR structure determination. The faster initial slope in case of **cp** geometry encodes shorter distances between ^1^H nuclei of NDC and DABCO (2.1 Å vs. 2.6 Å for **cp** and **op** forms, respectively), and thus reflects a more compact assembly architecture with respect to the **op** sample.

**Figure 2:**
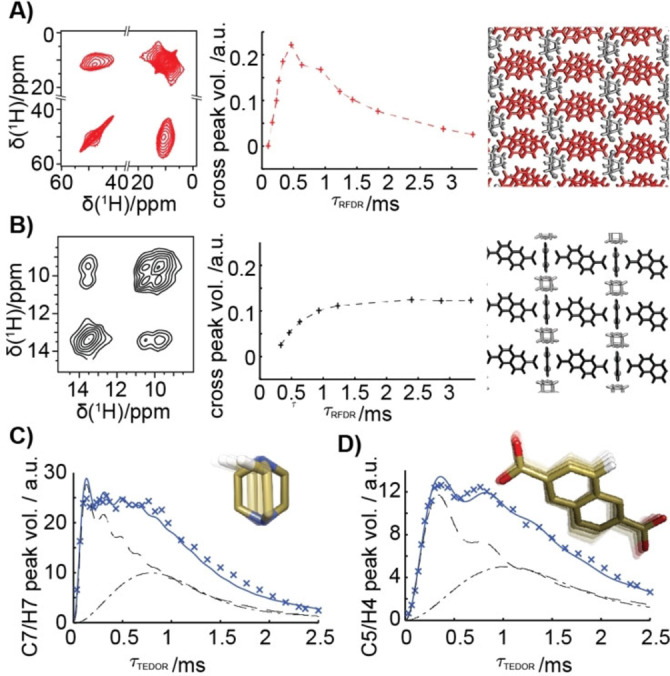
A,B) ^1^H–^1^H RFDR correlation spectra for **cp** (red) and **op** (black) DUT‐8(Ni) acquired with *τ*_RFDR_=0.3 ms, and H_DABCO_→H_NDC_ build‐up curves. Intensities were scaled to a maximum of diagonal H_DABCO_ signal equals one. Note that the accumulation of pulse imperfections (more pronounced for the broader spectrum of the **cp** sample) is responsible for the decrease observed for the build‐up curve of **cp** in *τ*_RFDR_>0.5 ms. C,D) ^1^H–^13^C TEDOR build‐up curves (×) for heteronuclear H7–C7 (C) and H4–C5 (D) correlations in the **op** DUT‐8(Ni). Data fit (solid line) was obtained using an analytical model consisting of two relaxation‐truncated Bessel functions (black lines;^[53]^ see SI for further details).

In parallel, the cross‐signal intensity in the TEDOR correlations is exquisitely sensitive to local dynamics, providing a picture which is independent of static distortions and disorder, hardly accessible with diffraction data. When the duration of the recoupling interval *τ*_TEDOR_ is incremented, the intensity of each correlation signal experiences a modulation whose frequency is proportional to the effective ^1^H–^13^C dipolar coupling constant. Such a constant can be extracted by directly fitting the oscillatory build‐up in the time domain, and order parameters *S*_ord_ can be calculated as a ratio between measured effective coupling values and those calculated from H and C distances in the X‐ray structure with DFT‐re‐optimised proton positions. Figure 2 C,D shows the experimental build‐up curves in the **op** form for a ^1^H–^13^C correlation within the NDC ligand (H4–C5) and one in the DABCO moiety (H7–C7; note that given the symmetry of the two organic groups, each correlation encodes a proximal and a distal ^1^H–^13^C dipolar coupling, see SI). By fitting these profiles, order parameters *S*_ord_ of 0.7 and 0.25, respectively, are obtained for the two spin pairs. The former corresponds to a highly restricted motion of the rigid NDC ligand,[^55^, ^56^] while the latter reports on very fast internal dynamics associated with the DABCO rotation around its N−N axis, in agreement with previous IR results,^[51]^ as well as with ^2^H MAS NMR and ^1^H *T*_1_ relaxation studies on analogous diamagnetic materials.[^57^, ^58^] This description of ligand dynamics is essential for understanding the tunability of guest‐adsorption properties.[^14^, ^15^, ^56^, ^59^] The different mobility of the NDC and DABCO ligands seems to be independent of the nature of the metal centre, as demonstrated by equivalent measurements on the analogue DUT‐8(Cu) **op** (see Section 9 of the SI).

The NDC and DABCO molecules are the key building blocks connecting the global structural features of the periodic framework during the pore‐closing process to a local deformation of the metal coordination geometry, and thus ultimately to the electronic fine structure of the PW node. In the following, we shed light on this connection, which has remained elusive despite tremendous efforts through the standard characterisation methods. The possibility to detect resolved paramagnetically shifted ^1^H signals indeed allows to measure their temperature dependence, which in turn is a direct reporter of the fine details of the electronic structure of the metal ions. The very recent development of MAS NMR probes capable of fast spinning at cryogenic temperatures[^60^, ^61^, ^62^] allows to extend the sensitivity benefits in a broad temperature range between 100 K and 305 K. In this way, MAS NMR can be used in a practical way to monitor the temperature dependence of resolved ^1^H shifts thus providing a microscopic insight into the magnetic properties of the system.

Magnetometric measurements and computational models revealed an antiferromagnetic coupling between the Ni^II^ centres in the **cp** form, resulting in a diamagnetic ground state ($S=\left|S_{1}-S_{2}\right|=0$ ) and in a thermal admixture of low‐lying paramagnetic excited states (S=1,2 ).[^51^, ^63^] As mentioned above, this configuration is mirrored in the smaller shift anisotropy of the ^1^H spectrum and the modest paramagnetic shift of the DABCO ^1^Hs. In line with this, we observed fundamental differences in the temperature behaviour of DABCO ^1^H NMR shifts in the two samples (Figure 3). While in the **cp** form the paramagnetic shift decreases with rising temperature in agreement with Curie law, in the **op** sample the shift has the opposite (“anti‐Curie”) temperature behaviour. The wide temperature range allows a quantitative analysis of the data with a simple Heisenberg model (see SI), which allows to extract both the Heisenberg coupling constant (*J*) within a Ni⋅⋅⋅Ni ion pair and the hyperfine coupling constant between Ni and DABCO ^1^Hs (*A*_M_).[^64^, ^65^] The fitted temperature dependence curves show an excellent agreement with the experimental data (RMSD=0.034 and 0.036 ppm for **cp** and **op**, respectively), despite some simplifications in the model, which notably neglects the contributions of pseudo‐contact shifts as well as hyperfine effects from distant metals.

**Figure 3:**
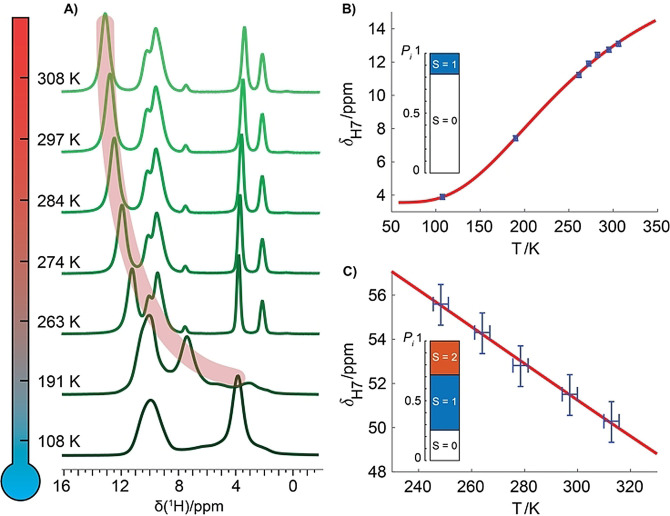
Temperature dependence H7 NMR shift of the A,B) **op** and C) **cp** DUT‐8(Ni) sample. In (A) ^1^H spectra (40 kHz MAS) of **op** DUT‐8(Ni) are reported, while in (B),(C) peak positions (blue+with error bars) are fitted by a Heisenberg model (red line). The bar‐plots indicate the Boltzmann population of electronic spin levels at 300 K for *J*_NiNi_=−266 and −52 cm^−1^ obtained from the fit in (B) and (C), respectively.

The value of the Ni⋅⋅⋅Ni Heisenberg coupling obtained for the **op** structure (*J*_**op**_=−266(13) cm^−1^ is significantly higher than thermal energy over the temperature range explored experimentally) explains the almost purely diamagnetic NMR shift (3.85 ppm) at cryogenic temperatures. The fitted value of *J*_**op**_ is in good agreement with the value determined through magnetometric measurements (−242 cm^−1^),^[51]^ and it matches the value calculated for a periodic crystal model by Seifert and co‐workers (−277 cm^−1^) as well.^[63]^ This reinforces the evidence of a high crystallinity for the **op** form. In contrast to magnetometric measurements,^[51]^ the temperature dependence of the ^1^H NMR shifts provides access to the Heisenberg coupling constant also for the **cp** form. Here, the experimentally fitted (*J*_cp_=−52(34) cm^−1^) value reveals a weaker Heisenberg coupling, in line with the longer Ni⋅⋅⋅Ni distance (2.653(5) Å and 2.735(9) Å for the **op** and **cp** structure, respectively),^[51]^ and with a more localised unpaired‐electron density (*A*_M_=1.4(2) and 1.0(4) for **op** and **cp**, respectively). Interestingly, the experimental |*J*_**cp**_| value is significantly lower than that calculated for a crystalline system (−108 cm^−1^), but fits very well with the one calculated for a truncated molecular Ni⋅⋅⋅Ni dimer model (−59 cm^−1^).^[63]^ This result suggests the presence of defects (i.e. the absence of DABCO units) in the **cp** form. These results demonstrate that ^1^H‐detected NMR provides the simultaneous elucidation of the bonding character and of the magnetic (super)exchange at the metal node, two essential elements for understanding phase transitions in switchable MOFs, both at a microscopic and macroscopic level.

In summary, we demonstrated a step forward in the analysis of paramagnetic MOFs, enabled by high MAS rates and carefully tuned pulse sequences. This allows to assign all proton and carbon resonances in paramagnetic DUT‐8(Ni), to determine local dynamics of the organic moieties next to the paramagnetic centre, and to disclose electronic properties of the nickel ions. For the first time the Heisenberg coupling of the closed‐pore form became experimentally accessible, providing unprecedented insight into switching behaviour and node deformation. The experiments described above constitute an important addition to the conventional repertoire of solid‐state NMR, notably allowing a reduction in sample quantities (often a bottleneck in the characterisation of new materials) and experiment times for recording NMR spectra and increasing the amount of information associated with them. Advantageously, with paramagnetic solid‐state NMR structural and electronic information can be obtained simultaneously under the same experimental conditions and on the same sample, thus avoiding any impact of different sample preparation on the measured properties and minimising the risk of sample degradation during multiple preparation steps. Due to the high gain in sensitivity, these methodologies will pave the way for elucidating the molecular structure of guest molecules, their interaction with the node and defects as well as resulting changes in the MOF properties in the future, also under in situ conditions, that is, during guest molecule adsorption.^[27]^ The simultaneous accessibility of this information is of utmost importance for understanding and tuning the materials response during catalytic or adsorption‐driven processes.

Finally, the methodologies are not limited to MOFs, but can also be implemented in the characterisation of other advanced materials containing paramagnetic centres, including silica materials or porous organic polymers.[^66^, ^67^, ^68^] They also disclose a wealth of new possibilities for the atomic‐level description of materials properties, and we anticipate that paramagnetic solid‐state NMR will cover in this context a bridging and highly complementary role between low‐resolution techniques reporting on bulk properties (such as magnetometry, gas sorption, and calorimetry) and those reporting on the periodic arrangement of the molecules (PXRD, electron and neutron diffraction).

## Conflict of interest

The authors declare no conflict of interest.

## Supporting information

As a service to our authors and readers, this journal provides supporting information supplied by the authors. Such materials are peer reviewed and may be re‐organized for online delivery, but are not copy‐edited or typeset. Technical support issues arising from supporting information (other than missing files) should be addressed to the authors.

Supporting Information
