# Supplementary material for: Monitoring Dynamics, Structure, and Magnetism of Switchable Metal–Organic Frameworks via 1H‐Detected MAS NMR
Source: Angew Chem Int Ed Engl. 2021 Sep 2;60(40):21778–83. doi: 10.1002/anie.202107032 (PMC8519119; doi:10.1002/anie.202107032)
Supplement: Supplementary file 1 — Supporting Information [file ANIE-60-21778-s001.pdf]

Angewandte Chemie
Eine Zeitschrift der Gesellschaft Deutscher Chemiker

Supporting Information

Monitoring Dynamics, Structure, and Magnetism of Switchable Metal–Organic Frameworks via ^{1}H-Detected MAS NMR

Jan Blahut, Arthur L. Lejeune, Sebastian Ehrling, Irena Senkovska, Stefan Kaskel, Florian M. Wisser, and Guido Pintacuda**

anie_202107032_sm_miscellaneous_information.pdf

Table of content

1. *Materials and Methods* 2
2. *Solid-state NMR* 3
3. *Exited-states population* 5
4. *TEDOR build-up fitting* 5
5. *Theoretical model* 6
6. *Linewidth analysis* 7
7. *Additional figures* 8
8. *Characterization of DUT-8(Ni)* 9
9. *Characterization of DUT-8(Cu)* 11
10. *References* 12

1. Materials and Methods

$Ni(NO_3)_2 \cdot 6H_2O$ (≥98%), 2,6-H_2ndc (99%), dabco, *N,N*-Diethylformamide (99%) and *N,N*-Dimethylformamide (>99.8%) were purchased from Sigma-Aldrich. Dichloromethane (DCM), and methanol (MeOH) purchased from commercial suppliers were of analytical grade and used without further purification. d^7-DMF (99.5% d) was purchased from Eurisotop. For the synthesis of DUT-8(Cu), 2,6-H_2-ndc was purified by dissolving in aqueous NaOH, filtration and re-precipitation of 2,6-H_2-ndc by acidifying the solution with concentrated HCl. All other chemicals were used as received.

DUT-8(Ni)

DUT-8(Ni) was synthesized according to the procedure given in Ref. [1] Typically, $Ni(NO_3)_2 \cdot 6H_2O$ (0.407 g, 1.40 mmol, 1.56 equiv) was dissolved in 6 ml DMF, 2,6-naphthalenedicarboxylic acid (2,6-H_2ndc, 0.303 g, 1.40 mmol, 1.56 equiv) was dissolved in 15 ml DMF and dabco (0.100 g, 0.89 mmol, 1 equiv) was dissolved in 9 ml methanol (MeOH). Subsequently the three clear solutions were mixed and transferred into a Teflon vessel (50 ml) and heated in an autoclave to 393 K for 48 hours. The resulting dark green crystals were washed several times with fresh DMF to obtain DUT-8(Ni)_*op*. For solvent removal, a part of the sample was soaked in DCM and the supernatant solution was replaced several times in the first two hours. After three days the crystals were filtered in argon atmosphere and the residual solvent was removed in dynamic vacuum at room temperature over night to obtain DUT-8(Ni)_*cp*. The sample was stored under inert atmosphere in a glove box until further use.

DUT-8(Cu)

DUT-8 (Cu) was synthesized by a slight modification of the literature procedure.[2]

In a 60 ml glass vial $Cu(NO_3)_2 \cdot 3H_2O$ (338 mg, 1.40 mmol. 1 equiv) was dissolved in 5 ml DEF. To the clear solution were added a solution of 2,6-H_2-ndc (303 mg, 1.40 mmol, 1 equiv) in 15 ml DEF (obtained by slightly heating the mixture in a glass vial) and a solution of dabco (79 mg, 0.70 mmol, 0.5 equiv) in 5 ml DEF. The glass vial was sealed and placed in an oven preheated at 120 °C for 24 h. After the reaction mixture was cooled to RT, the pale blue solid was isolated by centrifugation, the solvent exchanged to DMF. After soaking the MOF crystals for 2 h in DMF, the supernatant was removed by centrifugation and fresh DMF was added. The washing procedure was repeated four times. Activation first at RT then at 120°C for 24 h under vacuum yielded DUT-8 (Cu) as a pale blue solid. The sample was stored in an Argon-filled glovebox prior to MAS NMR studies.

Characterization methods

Scanning electron microscopy (**SEM**) measurements were carried out on a SU8020 from Hitachi using a 2 kV acceleration voltage and a working distance of 14.6 mm. Prior to the measurements, the samples were sputtered with Au to enhance the surface conductivity.

Powder X-ray diffraction (**PXRD**) experiments at room temperature of the solvated (**op**) and desolvated samples (**cp**) were carried out on an STOE STADI P diffractometer with Cu Kα1 radiation (λ = 1.5405 Å) and a 2D detector (Mythen, Dectris) in transmission geometry or on a a Bruker D5005 Diffractometer equipped with a Lyon-Eye detector in Bragg–Brentano geometry. DUT-8(Cu) samples were prepared inside an Argon filled glovebox and measured under a Kapton® foil to prevent sample degradation before measurement (auto sampler used).

For nitrogen physisorption at 77 K the sample was re-activated in dynamic vacuum at room temperature (DUT-8-(Ni)) for 16 hours or at 100 °C for at least 12 h (DUT-8(Cu)). The measurement was carried out either on a Quadrasorb SI apparatus from Quantachrome Company or on a BELSORP-mini II.

Thermogravimetric analysis was done using a Metter Toledo TGA/DSC 1 S**TA**R^e System under synthetic air (RT – 720 °C, heating rate: 2 K/min).

2. Solid-state NMR

Solid-state NMR experiments were performed on a Bruker Avance III spectrometer operating at a magnetic field strength of 16.4 T (corresponding to ^{1}H and ^{13}C Larmor frequencies of 700 and 176 MHz) using a Bruker 1.3 mm HCN narrow-bore MAS probe. Samples were packed into 1.3 mm zirconia rotors. Unless stated otherwise, all the solid-state experiments were performed at 60 kHz MAS (corresponding to a 16.67 µs rotor period) with the stator temperature regulated to 300 K corresponding to an approximate sample temperature of 325 K due to frictional heating. A ±5 °C temperature distribution within the sample was estimated from the width of the ^{207}Pb signal acquired on a crystalline $Pb(NO_3)_2$ sample under the same experimental conditions. 1D ^{1}H and ^{13}C experiments were acquired using spin-echo detection with 16.67 µs echo-time immediately following hard 90° excitation pulses (1.3µs@35W and 2.0µs@60W respectively). Longitudinal relaxation rates were measured using an adiabatic inversion recovery experiment and transverse relaxation rates were measured via double adiabatic echo experiments with variable, rotor-synchronized echo times. Short high-powered adiabatic tanh/tan pulses (SHAPs) were used to refocus the chemical shift evolution and invert population in order to avoid off-resonance effects.[3] Adiabatic pulses were 33.33 µs long and swept through 5 MHz with RF field strengths of 60 W and 35 W for ^{13}C and ^{1}H, respectively.
Deconvolution of spectral lineshapes to individual resonances were performed using a generalized-Lorentzian lineshape via the global spectra deconvolution toolbox included in MestReNova 12.0.3 software (Mestrelab). Analysis of rotational sideband patters was perfomed in the ssNake package with Haeberlen's definition of δ_{aniso} and η.[4]
The variable temperature experiments at temperatures ranging from 260 K to 300 K were performed employing the same equipment as described above, using the ^{1}H chemical shift of an external MeOH standard as a reference for temperature calibration.[5] The ultra-low temperature measurement were acquired on a Bruker Avance III spectrometer operating at a magnetic field strength of 18.8 T (corresponding to ^{1}H Larmor frequency of 800) using a MAS-DNP H/X/Y wide-bore probe with 1.3 mm zirconia rotor. In addition to the sample, the rotor contained ~1 mm^3 of crystalline KBr for internal temperature calibration via ^{79}Br T_1 relaxation.[6] All variable temperature measurements were performed at 40 kHz MAS frequency using a spin-echo detection experiment with a 25 µs echo-time. The temperature dependence of the experimental chemical shift was fitted using the Matlab R2018b (MathWorks) *fmincon* function minimalizing the root mean square deviation between the experimental NMR shift and its theoretical value obtained from equation (S1):

$$\delta = \delta^{\text{dia}} + A_M \frac{g_e \mu_B}{\hbar \gamma_{1\text{H}} 3 k_B T} \frac{\sum_{i=1}^{3} C_i S_i (S_i + 1)(2S_i + 1) \mathrm{e}^{-\frac{E_i}{k_B T}}}{\sum_{i=1}^{3} (2S_i + 1) \mathrm{e}^{-\frac{E_i}{k_B T}}} \qquad \text{(S1)}$$

where δ^{dia} is a diamagnetic contribution to the overall NMR shift, A_M is the hyperfine coupling between an observed ^{1}H nucleus and its closest metal, $\gamma_{1\text{H}}$ is the ^{1}H nuclear gyromagnetic ratio and other constants have their usual meaning. The parameter C_i corresponds to the contribution of single-metal spin-state ($s_{1,2}$) to the overall spin state S_i and it is tabulated.[7] For $s_{1,2}$=1, C_i equals to ½. The energy of each electronic level $E_i = -JS_i(S_i + 1)$ was calculated as based on an Heisenberg exchange interaction Hamiltonian $\hat{H}_{\text{ex}} = -2J\hat{\mathbf{s}}_1 \cdot \hat{\mathbf{s}}_2$, where J is a Heisenberg coupling constant.[8] The uncertainties of the parameters J and A were estimated as standard deviations of the parameters obtained by repeated (100×) fit of experimental data with random artificial error of ±2 K in temperature and of ±0.15ppm and ±1.5 pm in NMR shifts for **op** and **cp** respectively.
Homonuclear ^{1}H-^{1}H RFDR and heteronuclear ^{1}H-^{13}C HSQC-TEDOR correlation spectra were acquired according to the schemes described in ref. [9] and [10]. RFDR and TEDOR cross-peak build-up curves were obtained using the 2D integration tool in the TopSpin 4.0.7 software.

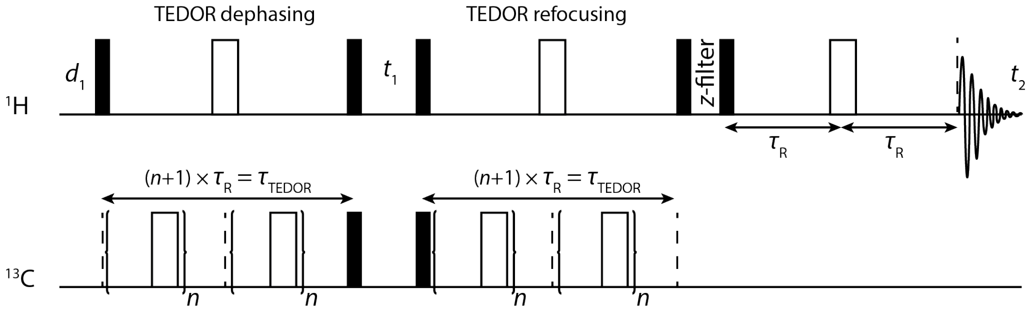

Figure S1: ^{1}H-detected HSQC-TEDOR pulse sequences. Filled and empty rectangles indicate 90° and 180° rectangular pulses respectively. τ_R is single-rotor-period time (16.66 μs at 60 kHz MSA). *z*-filter of 10 μs was used. Repetition delay d_1 of 0.1 s was used to ensure probe and amplifier duty-cycle. The d_1 was always at least ten-time longer then ^{1}H longitudinal relaxation time T_1 relaxation safely avoiding saturation. States-TPPI detection method was used for sampling of indirect dimension t_1 always with rotor synchronized t_1 increment 16.66 μs.

Assignment of ^{1}H and ^{13}C correlations to individual nuclei in DUT-8(Ni) samples is based on the combination of heteronuclear ^{1}H-^{13}C correlations observed after different τ_{TEDOR} recoupling times (Figure S2 A-D) and the analysis of the ^{1}H longitudinal relaxation rates. It exploits the geometrical dependence of the NMR effects and the knowledge of the organic components of the MOF. When a short τ_{TEDOR} recoupling period is employed, correlations can be observed exclusively over short ^{1}H-^{13}C distances, corresponding to a single bond (~1 Å). In the case of DUT-8(Ni) **op** form, the 2D correlation with τ_{TEDOR}=33.3 μs (Figure S2 A) resolves four signals, and the markedly different ^{1}H T_1s correlate with the Ni-^{1}H distances observed for H7, H4 and H1/H3 (Table S1). When longer τ_{TEDOR} recoupling periods are employed (Figure S2 B-D), correlations are observed over progressively longer distances, including between ^{1}Hs atoms and quaternary ^{13}Cs. Intermediate recoupling values (τ_{TEDOR}=133.3 μs, Figure S2 C) allow to span distances up to ~2.5 Å, and correlations over even longer distances (above 4 Å) are observed for very long values (2.5 ms, Figure S2 D). These build-ups corroborate the initial guesses.

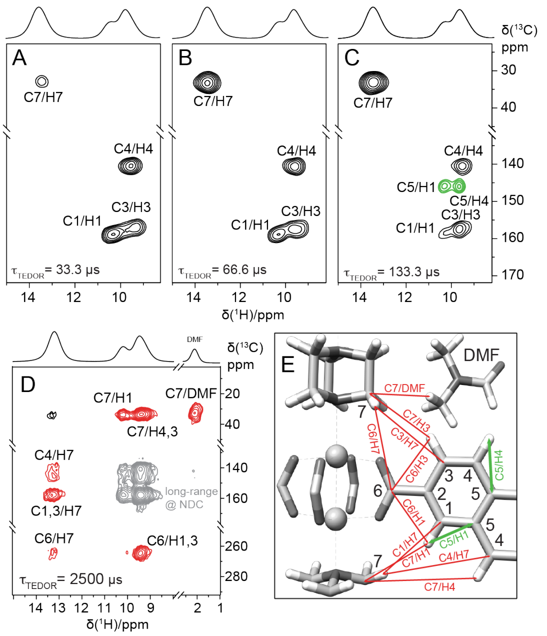

Figure S2: ^{1}H-^{13}C HSQC-TEDOR spectra of **op** DUT-8(Ni) for increasing recoupling time τREDOR: A) 33.3 μs, B) 66.6 μs, C) 133 μs and D) 2500ms. E) Scheme of the structure of the DUT-8(Ni) node highlighting the different H-^{13}C correlations observed.

3. Exited-states population

The populations P_i displayed in the inlet of figure 3 were calculated as follows:

$$P_i = \frac{N_i \exp\left(-\frac{E_i}{k_B T}\right)}{\sum_j N_j \exp\left(-\frac{E_j}{k_B T}\right)} \tag{S2}$$

where N_i is the degeneration of state i and other constants have their usual meaning.

4. TEDOR build-up fitting

Rotation-synchronized 180° pulses (each half-rotor-period) on the ^{1}H or ^{13}C channels reintroduce ^{1}H-^{13}C dipolar interactions in a spinning powder sample, leading to coherence transfer. The efficiency of the transfer depends on the orientation of each ^{1}H-^{13}C spin pair with respect to the axis of rotation, described with a polar angle β and an azimuthal angle α, and on the dipolar coupling constant D_{CH}. Each orientation contributes a transferred intensity s_D:

$$s_D = \sin^2\left(2\sqrt{2}\, D_{\mathrm{CH}} \sin 2\beta \sin\alpha\, \tau_{\mathrm{TEDOR}}\right) \tag{S3}$$

to the total TEDOR magnetization build-up S_D, which is in turn obtained by integration over all orientations in a powder solid:

$$S_D = \frac{1}{4\pi} \iint s_D \sin\beta \, \mathrm{d}\beta \mathrm{d}\alpha \tag{S4}$$

In our implementation the length of the coherence transfer and of the refocusing blocks are the same (τ_{TEDOR}) resulting in the total length of TEDOR pulse sequence of $2\tau_{\mathrm{TEDOR}}$. For a related formula for a REDOR dephasing curve[11] it was found that the solid-state integration can be replaced with a closed, analytical expression using Bessel functions.[12] Taking advantage of the fact that $\sin^2\alpha = \frac{1}{2}(1-\cos 2\alpha)$, the REDOR result can be used to obtain a closed expression for TEDOR magnetization transfer. Together with the refocused transverse dephasing times T_2', the build-up curve of TEDOR transferred intensity has the form

$$S_D = \left(\frac{1}{2} - \frac{\sqrt{2}\pi}{8} \mathcal{J}_{1/4}\left(2\sqrt{2} D_{\mathrm{CH}} \tau_{\mathrm{TEDOR}}\right) \mathcal{J}_{-1/4}\left(2\sqrt{2} D_{\mathrm{CH}} \tau_{\mathrm{TEDOR}}\right)\right) \exp\left(-\frac{2\tau_{\mathrm{TEDOR}}}{T_2'}\right), \tag{S5}$$

where $\mathcal{J}_a$ are Bessel functions of the first kind, $a = \pm 1/4$.

The dipolar coupling constants D_{CH} were fitted by minimizing the deviation between the experimental TEDOR build-up curve and the sum of the model build-up curves from Eq. (S3), with T_2' experimentally measured with a variable spin-echo (Table S1). The method yields D_{CH} with an accuracy of ±10%, as estimated based on repeated fits of the data with the addition of artificial noise and variation of T_2' rate within its conficence interval.

The dipolar coupling constants for a rigid model were calculated as

$$D_{\mathrm{CH}}^{\mathrm{rigid}} = \frac{\mu_0 \gamma_{13\mathrm{C}} \gamma_{1\mathrm{H}} \hbar}{8\pi^2 r} \tag{S6}$$

were γ_I are the gyromagnetic ratios of ^{1}H and ^{13}C and r their distance, obtained from a DFT refined X-ray structure.

5. Theoretical model

The geometrical structure for the calculation of rigid-model dipolar couplings was obtained by DFT refinement of a truncated DUT-8(Ni) OP X-ray structure (CCDC 1857107).[13] Cartesian coordinates of Ni, O and N atoms as well as C atoms of NDC were frozen during geometry optimization. The calculation was performed in Turbomole 7.1 with PBE0 functional[14,15] using Grimme's D3 dispersion correction[16] with Becke–Johnson damping.[17] Locally dense bases were employed – def2-TZVP for Cu and def2-SVP for the other atoms.[18] Calculations were performed *in vacuo* with overall charge 0 and pentet multiplicity. The following atomic coordinates (in Å) were obtained :

Ni	8.5088500	9.2040000	5.9498700
Ni	8.8384800	9.2040000	3.3265100
N	8.1720800	9.2040000	8.2708200
O	7.1424900	7.8031500	5.5676900
O	7.4232300	7.8068300	3.3218700
C	5.2018500	5.8445400	5.3812300
H	5.4814521	6.2354352	6.3608464
C	6.8751400	7.3981800	4.4025700
C	4.2133600	4.8468300	5.2708400
C	5.8273000	6.3323500	4.2634300
C	2.5811200	3.3539400	6.3042300
H	2.0683434	2.9457030	7.1769174
C	3.5251700	4.3148400	6.4415200
H	3.8031803	4.7019920	7.4248325
C	6.8121324	8.7209940	8.3804287
C	9.2245410	8.2864745	8.6619272
H	6.7380960	7.7314008	7.9124528
H	9.0614062	7.3144875	8.1783738
N	7.9465700	9.2040000	10.2819400
C	6.6591455	8.7345653	9.9086153
C	8.2161155	10.5849194	10.0848010
C	8.3864747	10.6082221	8.5593082
O	0.9168400	1.4008500	3.7087000
O	0.6360900	1.3971700	5.9545100
C	2.8574800	3.3594600	3.8951500
H	2.5695631	2.9629791	2.9195087
C	1.1841900	1.8058300	4.8738100
C	3.8459700	4.3571700	4.0055400
C	2.2320200	2.8716500	5.0129600
C	5.4782100	5.8500600	2.9721500
H	6.0027208	6.2691371	2.1126232
C	4.5341600	4.8891700	2.8348600
H	4.2557567	4.5012699	1.8520465
O	10.2048400	7.8031500	3.7087000
O	9.9240900	7.8068300	5.9545100
C	12.1454800	5.8445400	3.8951500
H	11.8628162	6.2334709	2.9156377
C	10.4721900	7.3981800	4.8738100
C	13.1339600	4.8468300	4.0055400
C	11.5200200	6.3323500	5.0129600
C	14.7662100	3.3539400	2.9721500
H	15.2790221	2.9457255	2.0994602
C	13.8221600	4.3148400	2.8348600
H	13.5441329	4.7019568	1.8515931
O	16.4304900	1.4008500	5.5676900
O	16.7112300	1.3971700	3.3218700
C	14.4898500	3.3594600	5.3812300
H	14.7777125	2.9628226	6.3568338
C	16.1631400	1.8058300	4.4025700
C	13.5013600	4.3571700	5.2708400
C	15.1153000	2.8716500	4.2634300
C	11.8691200	5.8500600	6.3042300
H	11.3475123	6.2708700	7.1647692
C	12.8131700	4.8891700	6.4415200
H	13.0920242	4.5016186	7.4243710
N	9.1752500	9.2040000	1.0055600
O	10.2048400	10.6048500	3.7087000
O	7.1424900	10.6048500	5.5676900
O	9.9240900	10.6011700	5.9545100
O	7.4232300	10.6011700	3.3218700
C	10.6128778	9.0789027	0.8162194
C	8.4539332	8.0752386	0.4414671
C	8.6772490	10.4753224	0.5043878
H	9.9090670	8.6765121	10.7365199
H	5.8749778	9.4182422	10.2704948
H	8.7678168	7.3174635	10.6042743
C	9.0345566	8.2997335	10.1827146
C	13.4706810	14.0560433	5.2638742
C	11.9296928	12.5009512	6.2799277
H	11.4727008	12.0424341	7.1580508
C	12.9039605	13.4869031	6.4046721
H	13.2248568	13.8152107	7.3961569
C	12.0920820	12.6467897	3.8723176
H	11.7580683	12.3016231	2.8925172
C	10.4721900	11.0098200	4.8738100
C	13.0629432	13.6360821	3.9975282
C	11.5200200	12.0756500	5.0129600
C	3.8776879	14.0557769	4.0084758
C	5.5542565	12.6342053	3.0115071
H	6.1192064	12.2792438	2.1484830
C	4.5833517	13.6233131	2.8852361
H	4.3733956	14.0606316	1.9062338
C	5.1204386	12.5137598	5.3871935
H	5.3496251	12.0651831	6.3552305
C	6.8751400	11.0098200	4.4025700
C	4.1463744	13.4996418	5.2595245
C	5.8273000	12.0756500	4.2634300
H	6.4632195	7.7404889	10.3413439
H	9.1212464	10.8882781	10.6346254
H	7.3800570	11.2028233	10.4493030
H	6.1405158	9.3939512	7.8287694
H	10.1918913	8.6649773	8.3054224
H	9.3793787	10.9072953	8.2009205
H	7.6482820	11.2076573	8.0083291
H	7.3829062	8.1770568	0.6575921
H	8.8035228	7.1440091	0.9067126
H	8.6009594	8.0112212	-0.6552680
H	10.8807477	9.0934083	-0.2589155
H	10.9612386	8.1402818	1.2655675
H	11.1227307	9.9048603	1.3297201
H	9.1875694	11.2987932	1.0220851
H	7.6039012	10.5601795	0.7156866
H	8.8455229	10.5711362	-0.5869122
H	14.2345851	14.8312941	5.3626212
H	13.5059645	14.0830465	3.1043590
H	3.1139471	14.8310065	3.9080937
H	3.5926668	13.8383025	6.1384601
H	-0.0160381	0.7186268	5.7153515
H	17.3632958	0.7185554	3.5610303

6. Linewidth analysis

Table S1: Comparison of ^{1}H NMR isotropic shift (δ_{iso} in ppm), full width at half-maximum intensity (FWHM in Hz), anisotropy of NMR shift (δ_{aniso} in kHz), its asymmetry (η) and longitudinal relaxation times (T_1), refocused transverse dephasing times (T_2') and transverse dephasing times (T_2^*) (in ms) acquired at 60 kHz MAS (700 MHz, 325 K sample temperature)

		δ_{iso}[e]	*FWHM*	δ_{aniso}[c]	η[c]	T_2*[d]	T_2'	T_1
**op**	NDC	9.3, 9.5[a]	~500[b]	-47	0.71	0.6	2.6(2)	9.4(1)
		10.3		-37	0.17		2.1(1)	6.7(1)
	DABCO	13.5	440	57	0.56	0.54	1.61(5)	2.69(4)
**cp**	NDC	9.5	3100	111	0.42	0.10	0.87(3)	3.4(1)
	DABCO	48.8	5600	-136	0.79	0.06	0.87(2)	1.21(4)

[a] Signals overlapped in 1D, δ_{iso} resolved in ^{1}H-^{13}C correlation see Figure 1. [b] average after deconvolution. [c] simulated via CSA pattern in ssNake package, (Figure S2) with Haeberlen definition of δ_{aniso} and η.[4] Note, that in this convention, sign of δ_{aniso} is often poorly defined for high values of η. [d] $T_2^* = 1/(\pi * FWHM)$.

Temperature distributions in the sample may contribute to the broadening of the signals. Specifically, a ±5 °C temperature distribution within the sample was estimated from the width of the ^{207}Pb signal acquired on a crystalline $Pb(NO_3)_2$ sample under the same experimental conditions. From the variable temperature spectra of figure 3, a slope of approx. 28 and –57 Hz/K can be estimated for **op** and **cp** respectively. The observed temperature distribution of ±5 K does thus not explain the ten-fold difference in LW between the two forms.

7. Additional figures

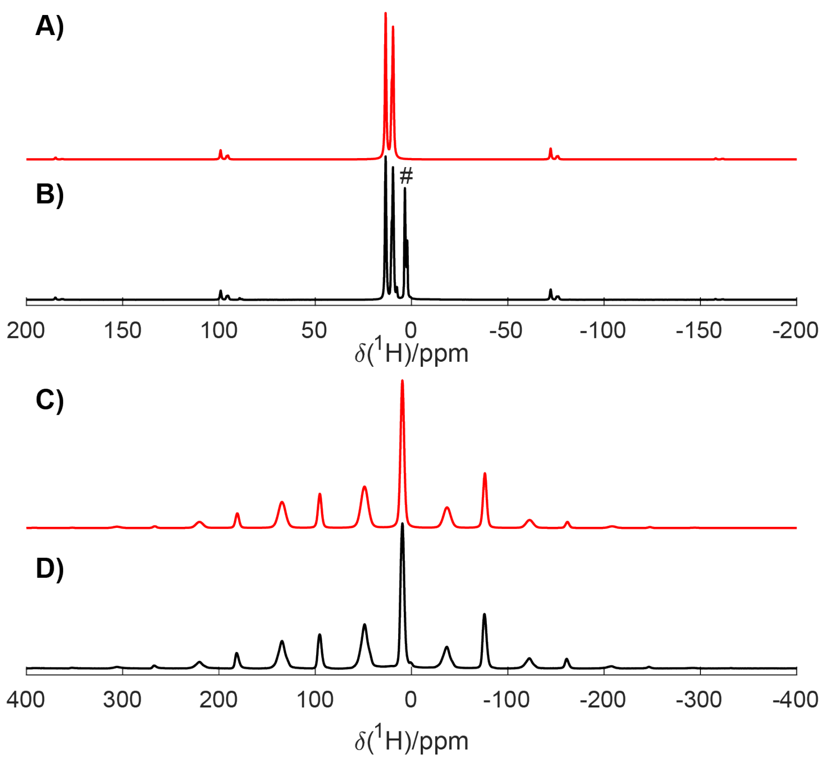

Figure S3: Solid-state MAS ^{1}H-NMR spectra of DUT-8(Ni) in **B**) **op** and **D**) **cp** forms obtained at 60 kHz MAS (700 MHz, 325 K sample. temp.) and the fit [**A**) and **C**)] of the rotation sideband pattern performed in ssNake package.[4] Obtained parameters listed in table S1. The hash mark (#) indicates residual signal of DMF.

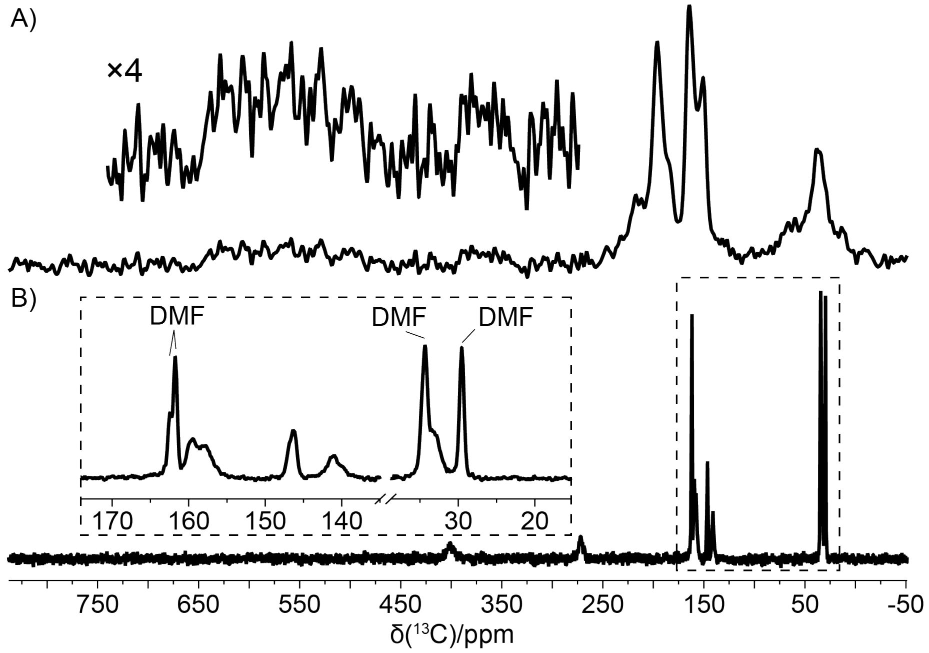

Figure S4: Solid-state MAS ^{13}C-NMR spectra of A) **cp** and B) **op** DUT-8(Ni) obtained with spin-echo detection of directly excited spectra. Interscan recycle delay d_1 was set to 0.1 and 0.4 s for **cp** and **op** sample respectively. (60 kHz MAS, 700 MHz, 325K sample. temp.)

8. Characterization of DUT-8(Ni)

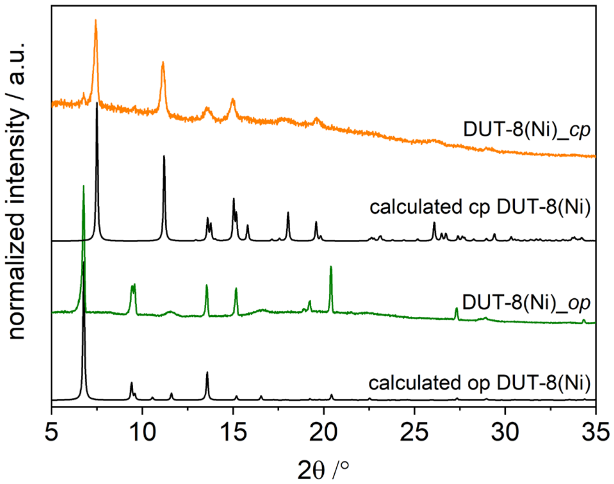

Figure S5: Powder XRD patterns of DUT-8(Ni) in the **op** phase (as made, green) and in the **cp** phase (desolvated, orange). The theoretical patterns were calculated from the corresponding crystallographic data (see CCDC 1857107 for **op** and CCDC 1034317 for the **cp**).[13,19]

The experimental pattern is typical for "as made DUT-8(Ni)" and confirms the phase purity and structural integrity of the sample.[13,20–22] The discrepancy between the experimental and calculated (reference) PXRD patterns observed in Figure S5 can be explained by two reasons. First, the calculated pattern of the open pore phase was generated from the idealized crystal structure of the framework with the empty pores. The experimental material contains (disordered) solvent molecules in the pores, contributing to the intensity of some experimental peaks. Second, the presence of specific guest molecules (here DMF) causes an intrinsic disorder of the linker molecules in DUT-8(Ni)_op. This disorder originates from the nonlinear character of the linkers arranging the clusters. More details can be found in our recent publication, reporting in detail the configurationally degenerate family of disordered states possible in DUT-8 and its influence on the PXRD patterns.[20] Note that the calculated PXRD pattern shown in Figure S5 represents only one borderline case (ordered monoclinic structure). Some discrepancies between the calculated and experimental PXRDs point on the discrepancies in the linker orientations in as made material in comparison to ordered monoclinic structure.

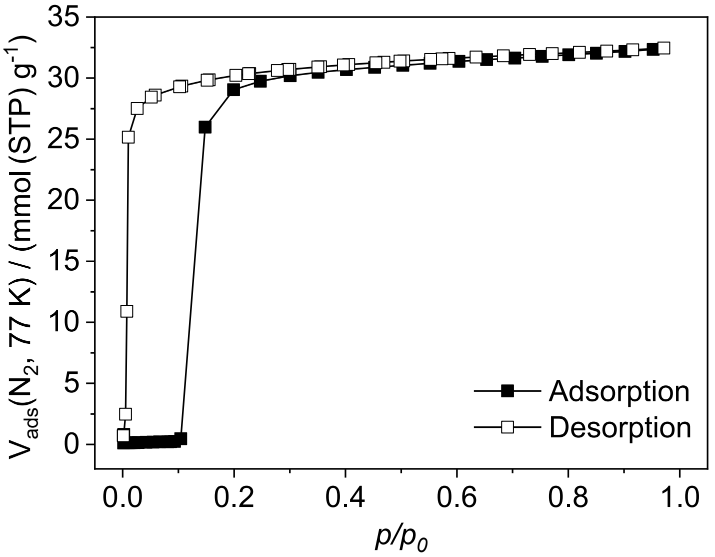

Figure S6: N_2 at 77 K physisorption isotherm of DUT-8(Ni) at 77K. Filled symbols - adsorption, empty symbols - desorption. Lines are traced uniquely for guiding the eyes.

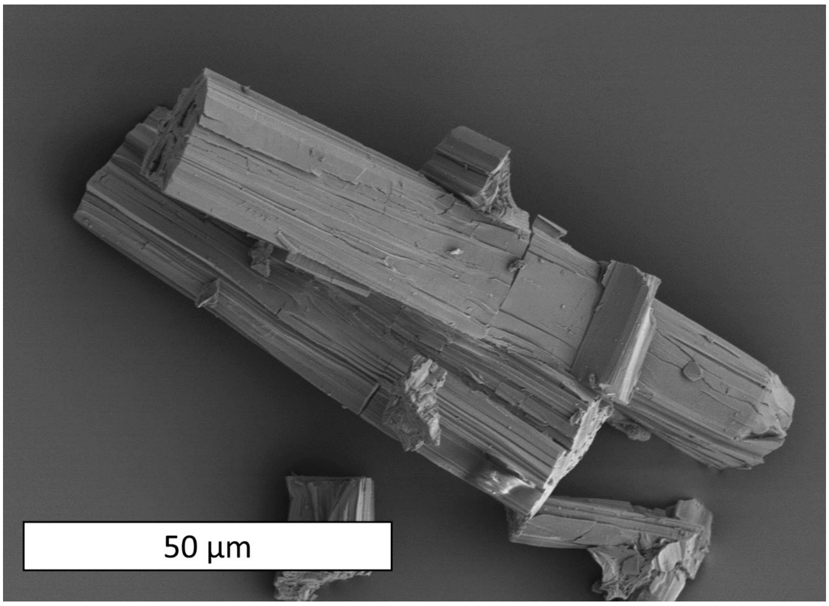

Figure S7: SEM image of DUT-8(Ni) **cp**. The magnification is displayed directly in the image.

9. Characterization of DUT-8(Cu)

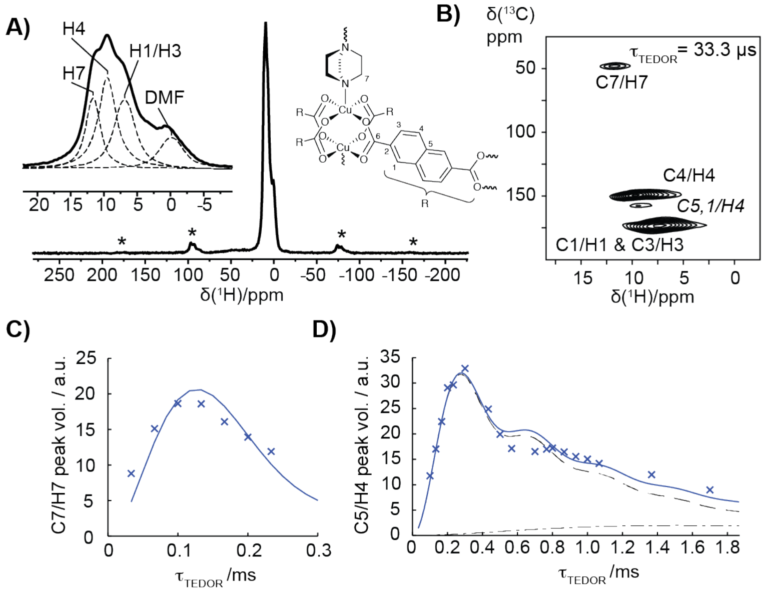

Figure S8: Solid-state MAS A) ^{1}H-NMR spectra of **op** DUT-8(Cu) acquired with rotor-synchronized spin-echo detection at 60 kHz MAS (700 MHz, 325 K of sample temperature) with an expansion of the spectral region of the isotropic resonances (the dashed line represents the deconvolution of the individual signals). B) ^{1}H-^{13}C HSQC-TEDOR acquired with short (33.3 µs) τ_{TEDOR} recoupling. C-D) ^{1}H-^{13}C TEDOR build-up curves (×) for heteronuclear H7-C7 C) and H4-C5 D) correlations in the **op** DUT-8(Cu). The best-fit models (solid lines) were calculated using an analytical model consisting of one C) and two D) relaxation-truncated Bessel functions of the dipolar constant. The obtained dipolar coupling constants correspond to order parameters S_{ord} of 0.19 and 0.87 for C) and D). Rotational sidebands and residual solvent signals are indicated by asterisks (*).

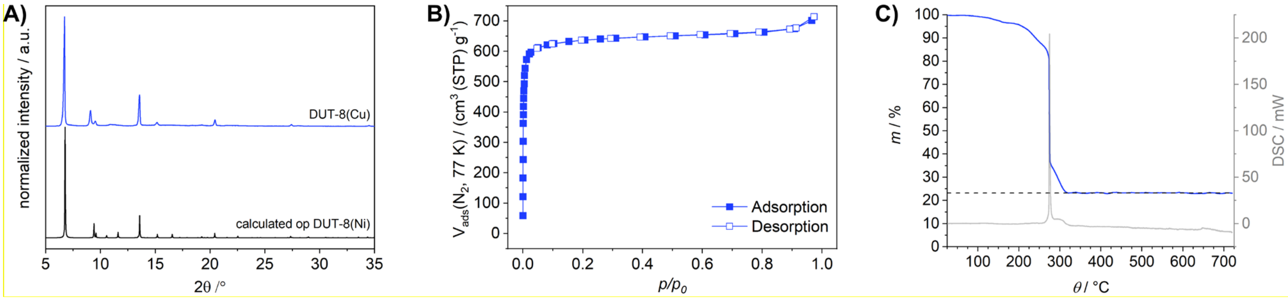

Figure S9: A) Powder XRD patterns of DUT-8(Cu) in the **op** phase (blue). The theoretical patterns were calculated from the corresponding crystallographic data (see CCDC 760964).[23] For differences in signal intensities between theoretical and measured PXRD pattern see section 8. B) N_2 at 77 K physisorption isotherm of DUT-8(Cu) at 77K. Filled symbols - adsorption, empty symbols - desorption. Lines are traced uniquely for guiding the eyes. C) TG curve (blue) for DUT-8(Cu) and corresponding DSC trace (gray). The dashed horizontal line corresponds to the expected amount of residual CuO for defect-free DUT-8(Cu).

10. References

[1] N. Kavoosi, V. Bon, I. Senkovska, S. Krause, C. Atzori, F. Bonino, J. Pallmann, S. Paasch, E. Brunner, S. Kaskel, *Dalt. Trans.* **2017**, *46*, 4685–4695.

[2] N. Klein, H. C. Hoffmann, A. Cadiau, J. Getzschmann, M. R. Lohe, S. Paasch, T. Heydenreich, K. Adil, I. Senkovska, E. Brunner, et al., *J. Mater. Chem.* **2012**, *22*, 10303.

[3] A. J. Pell, G. Pintacuda, *Prog. Nucl. Magn. Reson. Spectrosc.* **2015**, *84–85*, 33–72.

[4] S. G. J. van Meerten, W. M. J. Franssen, A. P. M. Kentgens, *J. Magn. Reson.* **2019**, *301*, 56–66.

[5] C. Ammann, P. Meier, A. Merbach, *J. Magn. Reson.* **1982**, *46*, 319–321.

[6] K. R. Thurber, R. Tycko, *J. Magn. Reson.* **2009**, *196*, 84–87.

[7] I. Bertini, C. Luchinat, G. Parigi, E. Ravera, in *Solution NMR of Paramagnetic Molecules*, Elsevier, **2017**, pp. 347–381.

[8] T. Soda, Y. Kitagawa, T. Onishi, Y. Takano, Y. Shigeta, H. Nagao, Y. Yoshioka, K. Yamaguchi, *Chem. Phys. Lett.* **2000**, *319*, 223–230.

[9] A. E. Bennett, R. G. Griffin, J. H. Ok, S. Vega, *J. Chem. Phys.* **1992**, *96*, 8624–8627.

[10] A. Bertarello, L. Benda, K. J. Sanders, A. J. Pell, M. J. Knight, V. Pelmenschikov, L. Gonnelli, I. C. Felli, M. Kaupp, L. Emsley, et al., *J. Am. Chem. Soc.* **2020**, *142*, 16757–16765.

[11] T. Gullion, in *Modern Magnetic Resonance* (Ed.: G.A. Webb), Springer Netherlands, Dordrecht, **2008**, pp. 713–718.

[12] K. T. Mueller, *J. Magn. Reson. Ser. A* **1995**, *113*, 81–93.

[13] P. S. Petkov, V. Bon, C. L. Hobday, A. B. Kuc, P. Melix, S. Kaskel, T. Düren, T. Heine, *Phys. Chem. Chem. Phys.* **2019**, *21*, 674–680.

[14] J. P. Perdew, K. Burke, M. Ernzerhof, *Phys. Rev. Lett.* **1996**, *77*, 3865–3868.

[15] J. P. Perdew, M. Ernzerhof, K. Burke, *J. Chem. Phys.* **1996**, *105*, 9982–9985.

[16] S. Grimme, J. Antony, S. Ehrlich, H. Krieg, *J. Chem. Phys.* **2010**, *132*, 154104.

[17] S. Grimme, S. Ehrlich, L. Goerigk, *J. Comput. Chem.* **2011**, *32*, 1456–1465.

[18] D. Rappoport, F. Furche, *J. Chem. Phys.* **2010**, *133*, 134105.

[19] V. Bon, N. Klein, I. Senkovska, A. Heerwig, J. Getzschmann, D. Wallacher, I. Zizak, M. Brzhezinskaya, U. Mueller, S. Kaskel, *Phys. Chem. Chem. Phys.* **2015**, *17*, 17471–17479.

[20] S. Ehrling, E. M. Reynolds, V. Bon, I. Senkovska, T. E. Gorelik, J. D. Evans, M. Rauche, M. Mendt, M. S. Weiss, A. Pöppl, et al., *Nat. Chem.* **2021**, *13*, 568–574.

[21] S. Ehrling, I. Senkovska, V. Bon, J. D. Evans, P. Petkov, Y. Krupskaya, V. Kataev, T. Wulf, A. Krylov, A. Vtyurin, et al., *J. Mater. Chem. A* **2019**, *7*, 21459–21475.

[22] S. Ehrling, M. Mendt, I. Senkovska, J. D. Evans, V. Bon, P. Petkov, C. Ehrling, F. Walenszus, A. Pöppl, S. Kaskel, *Chem. Mater.* **2020**, *32*, 5670–5681.

[23] N. Klein, C. Herzog, M. Sabo, I. Senkovska, J. Getzschmann, S. Paasch, M. R. Lohe, E. Brunner, S. Kaskel, *Phys. Chem. Chem. Phys.* **2010**, *12*, 11778–11784.
